# A Review of Omega-3 Fatty Acids from Marine Source Supplements and Enhanced Food Effects on Children’s Development, Neurological and Metabolic Disorders and General Health

**DOI:** 10.3390/md24040139

**Published:** 2026-04-15

**Authors:** Maria Dimopoulou, Stavroula Savvidi, Panagiotis Madesis, Aliki Dimopoulou, Dimitrios Stagos, Olga Gortzi

**Affiliations:** 1 Department of Agriculture Crop Production and Rural Environment, School of Agriculture Sciences, University of Thessaly, 38446 Volos, Greece; mdimopoulou@uth.gr (M.D.); pmadesis@uth.gr (P.M.); 2 Department of Biochemistry and Biotechnology, School of Health Sciences, University of Thessaly, Biopolis, 41500 Larissa, Greece; ssavvidi@uth.gr (S.S.); stagkos@med.uth.gr (D.S.); 3 Department of Psychology, School of Philosophy, National and Kapodistrian University of Athens, 15784 Athens, Greece; aliki_dimopoulou@hotmail.com; 4 POSS-Driving Innovation in Functional Foods PCC, Sarantaporou 17, 54640 Thessaloniki, Greece

**Keywords:** οmega-3 fatty acids, childhood, cognitive performance, learning outcomes, autism spectrum disorder, attention-deficit/hyperactivity disorder

## Abstract

Long-chain polyunsaturated fatty acids (LC-PUFAs) of omega-3 family, particularly docosahexaenoic acid and eicosapentaenoic acid, are essential nutrients that play a critical role in children’s growth and health. This review examines the evidence on the effects of omega-3 supplements and omega-3-enhanced foods on children’s development, as well as on neurological and metabolic disorders. Research consistently highlights the importance of DHA in brain and visual development, especially during early childhood, when rapid neural growth occurs. PubMed, Web of Science, Scopus and the Cochrane Library databases were searched for relevant articles published up to January 2026. Adequate omega-3 intake has been associated with improvements in cognitive performance, attention, and learning outcomes. In children with neurodevelopmental conditions such as attention-deficit/hyperactivity disorder and autism spectrum disorder, omega-3 supplementation shows modest but potential benefits in reducing behavioral symptoms and supporting executive function, although results remain mixed. Additionally, omega-3 fatty acids exhibit anti-inflammatory properties that may positively influence metabolic health, including lipid profiles, insulin sensitivity, and obesity-related risk factors in children. Omega-3-enhanced foods provide an alternative to supplements and may improve adherence and overall dietary quality. However, variability in dosage, study design, and baseline nutritional status limits definitive conclusions. Overall, omega-3 fatty acids appear to support healthy development and may aid in managing certain neurological and metabolic disorders in children.

## 1. Introduction

Optimal nutrition during childhood is a fundamental determinant of growth, neurodevelopment, metabolic health, and long-term disease risk [1]. Among essential nutrients, omega-3 polyunsaturated fatty acids (PUFAs) have received substantial scientific attention due to their critical structural and functional roles in the developing brain, immune system, and metabolic pathways [2]. Omega-3 fatty acids cannot be synthesized de novo in sufficient quantities by the human body and must therefore be obtained through diet or supplementation [3]. The most biologically active omega-3 fatty acids are eicosapentaenoic acid (EPA) and docosahexaenoic acid (DHA), which are predominantly derived from sources such as fish, seafood fish oils and alga-based products [4].

Childhood represents a period of rapid cellular growth, brain maturation, and metabolic programming, during which nutritional inadequacies may have lasting consequences [3]. DHA, in particular, is a major structural component of neuronal membranes and retinal photoreceptors, accounting for a significant proportion of total fatty acids in the cerebral cortex and synaptic membranes [5]. Accumulation of DHA in the brain begins during the third trimester of pregnancy and continues throughout infancy and early childhood, coinciding with periods of synaptogenesis, myelination, and neural circuit refinement [6]. Consequently, insufficient intake of marine omega-3 fatty acids during these critical windows may adversely affect neurodevelopmental outcomes, cognitive function, and behavioral regulation.

Despite the biological importance of omega-3 fatty acids, dietary intake among children remains suboptimal worldwide, particularly in regions with low fish consumption or limited access to omega-3-rich foods [7]. Modern dietary patterns characterized by high consumption of processed foods and excessive omega-6 fatty acids may further exacerbate omega-3 deficiencies by altering fatty acid balance and inflammatory pathways [3]. These trends have fueled interest in omega-3 supplementation and food fortification strategies as potential public health interventions to support child health and development [8].

Omega-3 fatty acids exist in several forms, including alpha-linolenic acid (ALA), found in plant sources such as rapeseed oil (as a major consumed oil which contains appreciable ALA), flaxseed and walnuts, and LC-PUFAs EPA and DHA, found primarily in aquatic sources [9]. Although ALA can be endogenously converted to EPA and DHA, conversion rates are extremely limited—often less than 10% for EPA and below 1% for DHA—especially in children [10]. As a result, direct intake of marine-derived EPA and DHA is considered nutritionally superior for meeting physiological requirements [11].

LC-PUFAs are available through natural dietary sources (fish and seafood), dietary supplements (fish oil, krill oil, and algal oil), [12] and omega-3-enhanced or -fortified foods such as infant formula, dairy products [13], eggs [2] and spreads [14]. Enhanced foods have been developed to address low intake in populations with limited fish consumption, particularly among children who may have dietary preferences or restrictions [15]. EPA and DHA occur in foods and supplements predominantly in esterified (bound) forms rather than as free fatty acids. In marine fish oils, such as those derived from *Engraulis ringens* and *Salmo salar*, EPA and DHA are mainly esterified to triacylglycerols (TAGs) [16]. In contrast, Antarctic krill oil from *Euphausia superba* contains a substantial proportion of EPA and DHA in phospholipid form, particularly phosphatidylcholine [17]. Marine microalgae, the primary producers of long-chain omega-3 PUFAs, also differ in lipid class distribution: in *Schizochytrium* sp. and *Crypthecodinium cohnii*, DHA is largely accumulated in TAGs, whereas in *Phaeodactylum tricornutum* and *Nannochloropsis gaditana*, EPA is enriched in glycolipids and phospholipids associated with cellular membranes [18]. During digestion, pancreatic lipase and phospholipase A_2_ hydrolyze these lipid classes into 2-monoacylglycerols and lysophospholipids, which are incorporated into mixed micelles and absorbed by enterocytes [19].

Human and animal studies comparing lipid forms demonstrate that EPA and DHA from phospholipids or re-esterified TAGs can show equal or, in some cases, enhanced absorption compared with ethyl ester forms, particularly under low-fat meal conditions. Clinical trials using DHA-rich oil from *Schizochytrium* sp. report efficient incorporation of DHA into plasma phospholipids and erythrocyte membranes, comparable to fish-derived TAG sources [20]. Similarly, EPA from *Nannochloropsis gaditana* biomass has demonstrated good bioaccessibility after simulated digestion and measurable increases in circulating EPA in human supplementation studies [21]. These findings support the concept that plant-based (microalgal) long-chain omega-3 PUFAs, when consumed in natural lipid matrices (TAGs, glycolipids, or phospholipids), are effectively digested and absorbed. Recent work employing stable isotope tracers and postprandial lipidomics further confirms that the structural form of EPA and DHA modulates kinetics of absorption and tissue distribution, but overall bioavailability from well-formulated microalgal sources is high, reinforcing their suitability as sustainable alternatives to marine fish oils [20].

Global dietary surveys indicate that omega-3 intake among children is often below recommended levels [22], especially in populations with low fish consumption [7,23]. This deficiency has raised concerns regarding its potential impact on neurodevelopment, cognitive performance, immune function, and metabolic health. In recent decades, increasing attention has been given to omega-3 supplementation and fortified foods as strategies to improve omega-3 status and support child health outcomes [5]. Despite extensive research in adults, the effects of omega-3s on children’s developmental and health outcomes remain inconsistent across studies, with many contradictory findings and insufficient synthesis of evidence from diverse age groups and health contexts, partly due to the predominance of short-term studies [24]. However, the need for a comprehensive evaluation of omega-3 effects on children’s development, neurological and metabolic disorders, and general health provides the rationale for the present review. There is sufficient accumulated clinical and observational evidence to justify this review. Importantly, the heterogeneity and inconsistency in findings make a comprehensive, structured synthesis not only feasible but necessary.

## 2. Results and Discussion

Given the critical role of omega-3 LC-PUFAs in childhood development, the variability in dietary intake, and the growing body of clinical research, a comprehensive review of the current evidence is warranted. This review aims to synthesize findings from observational studies, RCTs, and systematic reviews on the effects of omega-3 fatty acids from marine sources, supplements, and enhanced foods [25,26,27,28] on children’s development [25,29,30,31,32,33,34,35,36,37], neurological/behavioral [38,39,40,41,42,43,44,45,46,47,48,49,50,51,52,53,54] and metabolic disorders [28,55,56,57,58,59,60,61,62,63,64,65], and general health [66]. By identifying areas of consensus and gaps in knowledge, this review seeks to inform clinical practice, nutritional guidelines, and future research directions.

Below are tables summarizing key randomized controlled trials (RCTs) and dosage ranges of marine-derived omega-3 fatty acids (EPA/DHA) in children, organized by developmental, neurological, metabolic, and general health outcomes.

### 2.1. Effects on Child Growth and Neurodevelopment

#### 2.1.1. Childhood Cognitive Development

**Figure 1:**
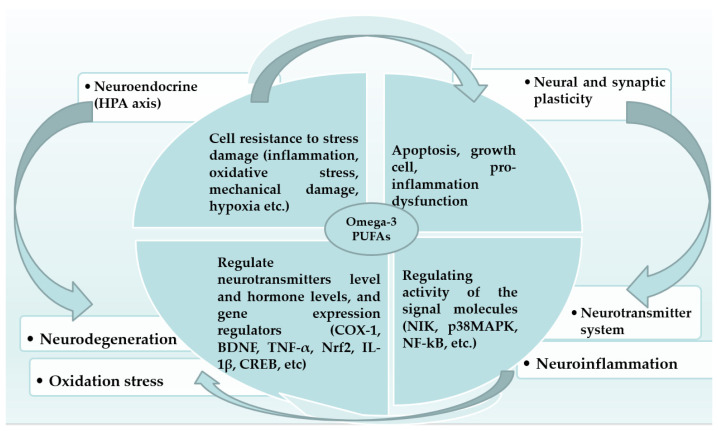
Key mechanisms of omega-3 PUFAs in the central nervous system include anti-inflammation action, structure role and membrane fluidity, neuroprotection and antioxidant effects, synaptic plasticity and neurogenesis, and gut–brain axis modulation (COX-1: cyclooxygenase 1; BDNF: brain-derived neurotrophic factor; TNF-a: tumor necrosis factor a; Nrf2: nuclear factor erythroid 2-related factor 2; IL-1β: Interleukin-1 beta; CREB: cAMP response element-binding protein; NΙΚ: NF-κB-inducing kinase; p38MAPK: p38 mitogen-activated protein kinase; NF-KΒ: nuclear factor kappa-B) [78].

**Figure 2:**
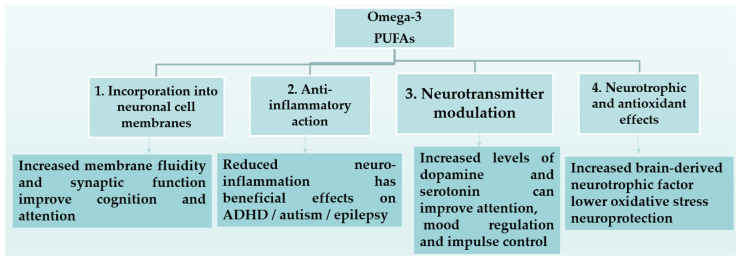
Omega-3 PUFA consumption provides neuroprotection and has beneficial effect on neurological disorders during childhood [79].

DHA is a major structural component of neuronal membranes and supports neurogenesis, synaptogenesis, migration, and neurotransmission—processes that are critical during rapid growth phases like infancy and adolescence, while EPA modulates neuroinflammation and cerebral blood flow (Figure 1) [67]. Experimental studies demonstrate that omega-3 deficiency during early life impairs synaptic plasticity, alters dopamine and serotonin signaling, and increases vulnerability to neuroinflammatory damage (Figure 2) [68,69]. IQ scores of children who were fed a formula containing either LC-PUFAs or no LC-PUFAs did not differ at age 6 year. However, children who received LC-PUFAs were faster at processing information compared with children who received unsupplemented formula [37]. LC-PUFAs are also critical for retinal development [70] and visual acuity, especially in preterm infants [25,29,30,31]. Met analyses note that LC-PUFA supplementation may yield modest improvements in executive function, attention, working memory, and processing speed in children, but effects are often small and inconsistent across trials [25,26]. Benefits seem more likely when baseline omega-3 status is low and intakes exceed ~450 mg EPA and DHA per day in children [33,34] and adolescents [34,35], suggesting a potential threshold effect for cognitive efficacy. Overall, evidence indicates short-term neurocognitive impacts in some domains (e.g., visual attention and working memory), but these changes may not persist long term without sustained intake (Table 1). The greatest effects are observed in visual/retinal development in early life and in processing and memory in adolescents with low baseline omega-3 status. According to Cohen’s d effect size, most effects were small, with the exception of visual/retinal and psychomotor for pregnancy to infancy but also processing speed and memory for adolescents (10–16 years) [25,29,30,31,32,33,34,35,36,37].

**Table 1:** Key randomized controlled trials of marine omega-3 fatty acids on neurodevelopment and cognition in children.

Age Group	Participants (*n*)	Source	Dose	Duration	Main Outcomes	Key Findings	References
Pregnancy → infancy	*n* = 200–1200	Fish oil	200–800 mg DHA/day	Pregnancy and infancy	Cognitive and visual development	Improved visual acuity and early psychomotor development; inconsistent long-term IQ effects	[25,29,30,31,36]
Infants (0–12 months)	*n* = 150–600	DHA-fortified formula	0.2–0.35% FA	6–12 months	Visual acuity; MDI	Improved retinal and visual outcomes; mixed cognitive effects	[25,26,37]
6–12 years	*n* = 120–400	Fish oil	300–600 mg EPA + DHA/day	3–6 months	Attention; executive function	Modest improvements in attention and working memory	[33,34]
10–16 years	*n* = 100–250	Fish oil	≥450 mg EPA + DHA/day	12–24 weeks	Processing speed; memory	Greater benefits in individuals with low baseline omega-3 status	[34,35]

#### 2.1.2. Behavioral and Psychiatric Conditions

Omega-3 status and supplementation have been studied extensively in children with neurodevelopmental disorders [70]. Children with attention-deficit/hyperactivity disorder (ADHD) frequently exhibit lower blood levels of DHA and EPA compared to typically developing peers [71]. Across four study groups (Table 1) comprising approximately 570–2450 participants, omega-3 supplementation was associated with modest improvements in attention and cognitive outcomes, particularly among individuals with low baseline omega-3 status. Across seven randomized clinical trials (Table 2) [38,39,40,41,42,53,72] including 511 participants, lower omega-3 fatty acid status was consistently associated with greater symptom severity in domains such as inattention and hyperactivity; however, due to the lack of reported paired quantitative data, a precise correlation coefficient could not be calculated. Only one study reported a significant association between reductions in fatty acid levels and decreases in hyperactivity, but without providing a numerical correlation value.

Meta-analyses of RCTs highlight small but statistically significant improvements in ADHD symptoms and attention performance following marine LC-PUFA supplementation, especially in studies with higher doses of EPA and over longer durations. Small-to-moderate improvement in attention and behavior has been found with 300–1000 mg/day of EPA-dominant in children with ADHD [71,73]. Some trials suggest efficacy when LC-PUFAs are used adjunctively with standard pharmacotherapy, but the magnitude of benefit varies and is often modest, reinforcing that omega-3s are supportive rather than curative [39,40,41,42,74]. However, although the magnitude of effect is generally modest compared to stimulant medications, omega-3s are well tolerated and may offer benefits for children who experience medication side effects or have suboptimal dietary intake.

As for autism spectrum disorder (ASD), observational studies show lower omega-3 PUFA levels in children with ASD relative to controls, with potential links to behavior and social functioning [48]. RCTs of LC-PUFA supplementation in ASD have produced mixed results, with some small pilot studies reporting modest behavioral improvements and others finding no significant benefit on core ASD symptoms. Longitudinal and larger RCTs are needed to clarify efficacy. Emerging evidence suggests omega-3 may also influence the gut–brain axis, potentially improving microbiota diversity and reducing systemic inflammation, mechanisms relevant to ASD symptomatology, though this is an evolving research area [75]. The heterogeneity of ASD, combined with small sample sizes and variable dosing regimens, complicates interpretation and underscores the need for larger, well-designed trials.

Limited studies indicate potential beneficial effects of LC-PUFA supplementation on dyslexia and developmental coordination disorders (DCDs) [76], with some improvements in academic skills and motor coordination when combined with other nutrients, but research remains preliminary and requires further validation [76]. PCSO-524^®^ (Lyprinol/Omega-XL), a marine lipid extract from the New Zealand green-lipped mussel (*Perna canaliculus*), has been studied in children aged 6–14 years for ADHD symptoms (e.g., hyperactivity and inattention), though not specifically for DCD; ADHD may be relevant due to overlapping neurodevelopmental symptom profiles. A large RCT investigated PCSO-524^®^ vs. placebo in children and adolescents with high hyperactivity/inattention. Some subgroup analyses suggested improvements in attention, learning and hyperactivity, although these effects were not sustained across all outcomes. This trial did not include a DCD-diagnosed sample, though research protocols highlighted the anti-inflammatory and omega-3-rich profile of the extract derived from a marine source [77]. Finally, diet patterns such as the Mediterranean diet combined with LC-PUFA supplementation have been associated with reduced impulsive behavior in children with ADHD [38]. With regard to neurodevelopmental disorders during childhood and adulthood, 18 RCTs were included in this review (Table 2) [38,39,40,41,42,43,44,45,46,47,48,49,50,51,52,53,54,72].

**Table 2:** Summary of randomized clinical trials of LC-PUFA supplementation in neurodevelopmental disorders during childhood and adulthood.

Study (Year)	Age Group	Participants (*n*)	Disorder	Dose (EPA + DHA)	Duration	Main Outcomes	Key Findings	References
San Mauro Martin et al. (2022)	10–12 years	*n* = 60	ADHD	550 mg EPA and 225 mg DHA	8 weeks	Attention; hyperactivity scales	Less marked effects; associated with reduced impulsive behavior in children with ADHD, with improved behavioral outcomes when combined with a Mediterranean diet	[38]
Carucci et al. (2022)	6–12 years	*n* = 160	ADHD	Two capsules each containing 279 mg EPA, 87 mg DHA, and 30 mg gamma-linolenic acid (GLA)	12 months	No effect was found on mood and anxiety symptoms; improvement in reading and writing difficulties global functioning and motor abilities	Limited role of omega-3/6 dietary products in children with mild ADHD	[39]
Barragán et al. (2017)	5–15 years	*n* = 90	ADHD	Omega-3/6 fatty acids (Equazen Eye Q™, Vifor Pharma UK Limited, Wigan, UK) with methylphenidate (MPH) and combined MPH + omega-3/6	12 months	Adverse events were numerically less frequent with omega-3/6 or MPH + omega-3/6 than with MPH alone	Hyperactivity and impulsivity	[40]
Assareh et al. (2017)	6–12 years	*n* = 40	ADHD	241 mg DHA, 33 mg EPA, and 180 mg omega-6 (Minami Company, Kontich, Belgium) once daily	10 weeks	The results did not support the efficacy of PUFAs in the treatment of ADHD	Improvement in inattention, hyperactivity, and impulsivity with methylphenidate but not differences with PUFA supplementation	[41]
Bos et al. (2015)	8–4 years	*n* = 39	ADHD	10 g of margarine daily, enriched with either 650 mg of EPA/DHA or placebo	16 weeks	Reduction in ADHD symptoms in both individuals with ADHD and typically developing children	Reduction in attention problems, rule breaking behavior and aggressive behavior	[42]
Berger et al. (2026)	15.7 years	*n* = 257	MDD	1.5 g/day (1 g EPA and 0.5 g DHA; 2:1 ratio)	36 weeks	No differences in symptom trajectories, remission and response rates, additional antidepressant use, or quality of life measures between control and intervention groups	No statistically significant benefit	[43]
Gabbay et al. (2018)	12–19 years	*n* = 51	MDD	Initial dose of 1.2 g/day, increased by 0.6 g/day every 2 weeks, up to a maximum of 3.6 g/day	6 months	Not superior on any clinical feature, including depression severity and levels of anhedonia, irritability, and suicidality	Both treatments associated with significant improvement in depression severity	[44]
Häberling et al. (2019)	8–17 years	*n* = 220	MDD	1.5 g/day; in children under 13 years old, half the dose administered, resulting in 500 mg EPA and 250 mg DHA per day	36 weeks	Absence of major depression for >4 months, as well as adverse remission and recovery rates	Antidepressant properties; increased markers of oxidative stress, and/or markers of (low grade) inflammation	[45]
Fristad et al. (2015)	7–14 years	*n* = 23	Bipolar Disorder	Two 500 mg omega-3 capsules (350 mg EPA, 50 mg DHA and 100 mg other omega-3 fatty acids) twice daily, for a total daily dose of 2000 mg of omega-3 (1400 mg EPA, 200 mg DHA and 400 mg other omega-3 fatty acids)	12 weeks	Combined therapy associated with greater improvement in depressive symptoms	Decreased manic and depressive symptoms; improved global functioning	[46]
Katrenčíková et al. (2021)	7–18 years	*n* = 60	Depressive disorder compared to healthy controls	Fish oil emulsion consisted of 2.4 g total omega-3 fatty acids (1.0 g EPA and 0.75 g DHA; EPA:DHA ratio = 1.33:1)	12 weeks	No differences observed in SOD and CAT activities or TEAC between children with depression and healthy controls; significant negative correlations found between CDI and TEAC, SOD and GPx, respectively	Oxidative stress may be associated with the severity of depression in children and adolescents; LC-PUFA supplementation may influence oxidative stress markers	[47]
Mazahery et al. (2019)	2.5–8 years	*n* = 73	ASD	722 mg DHA with 2000 IU vitamin D3	12 months	Behavior and social responsiveness	Mixed results; modest behavioral improvements in some trials	[48]
Richardson et al. (2005)	5–12 years	*n* = 117	DCD	6 capsules provided omega-3 fatty acids (558 mg EPA and 174 mg DHA) and the omega-6 fatty acid linoleic acid (60 mg), plus 9.6 mg vitamin E (natural form, α-tocopherol)	3 months	Motor coordination and reading; improvements with active treatment versus placebo in reading, spelling and behavior	Safe and efficacious treatment option for educational and behavioral problems in children with DCD; improved motor skills and academic performance	[76]
Nemets et al. (2006)	6–12 years	*n* = 28	Mood and emotional regulation	1000 mg/day	12–16 weeks	Mood and anxiety scales	Preliminary benefit; limited pediatric trials	[50]
Fristad et al. (2019)	7–14 years	*n* = 72	Major depression, dysthymia, or depression	Two 500 mg omega-3 capsules (350 mg EPA: 50 mg DHA, a 7:1 ratio; 68 mg other omega-3) twice daily for a total daily dose of 1870 mg omega-3	12 weeks	Depression	Relative to placebo, youth with fewer social stressors responded better to omega-3 and their combination with psychoeducational psychotherapy; small to medium effects of combined treatment and omega-3 monotherapy to depression	[51]
Wang et al. (2025)	13–24 years	*n* = 51	Depression	Fish oil supplementation (2700 mg/day of ω3 PUFAs, including 1941 mg of EPA and 759 mg of DHA)	12 weeks	Depression	Omega-3 PUFAs promoted phospholipid integration and alleviated oxidative stress, which may account for their antidepressant effects	[52]
Widehorn-Müller et al. (2014)	6–12 years	*n* = 95	ADHD	A daily dose of 720 mg omega-3 fatty acids (600 mg EPA, 120 mg DHA) and 15 mg of vitamin E as antioxidant	16 weeks	Association between erythrocyte fatty acid composition and behavior and cognitive function	Supplementation with the omega-3 fatty acid mix increased EPA and DHA concentrations in erythrocyte membranes and improved working memory function, but had no effect on other cognitive measures and parent- and teacher-rated behavior in the study population	[53]
Parellada et al. (2017)	5–17 years	*n* = 68	ASD	Omega-3 (962 mg/day and 1155 mg/day for children and adolescents, respectively) (EPA + DHA, 33% + 22% of the total daily fish oil supplemented) and vitamin E as a stabilizer	8 weeks	Omega-3 supplementation improves erythrocyte membrane omega-6/omega-3, plasma antioxidant status (TAS) and autistic behaviors	Improvement in Social Motivation and Social Communication subscale scores, with a moderate to large effect size (*p* = 0.004, day = 0.73, and *p* = 0.025, day = 0.79, respectively), but no treatment effect (treatment-placebo order)	[54]
Bent et al. (2011)	3–8 years	*n* = 27	ASD	Orange-flavored pudding packets (Coromega^®^, Vista, CA, USA) containing 650 mg of omega-3 fatty acids, including 350 mg of EPA and 230 mg of DHA, given twice daily for a daily dose of 1.3 g of omega-3 fatty acids (and 1.1 g of DHA + EPA)	12 weeks	Aberrant Behavior Checklist	Hyperactivity, as measured by the Aberrant Behavior Checklist, improved 2.7 (±4.8) points in the omega-3 group compared to 0.3 (±7.2) points in the placebo group (*p* = 0.40; effect size = 0.38); correlations were found between decreases in five fatty acid levels and decreases in hyperactivity, and the treatment was well tolerated	[72]

ADHD: attention-deficit/hyperactivity disorder; ASD: autism spectrum disorder; MDD: major depressive disorder; DCD: developmental coordination disorder; PUFAs: polyunsaturated fatty acids; DHA: docosahexaenoic acid; EPA: eicosapentaenoic acid; ABC: Aberrant Behavior Checklist; TEAC: Trolox equivalent antioxidant capacity of serum; Cu/Zn SOD: copper/zinc superoxide dismutase; GPx: glutathione peroxidase; CAT: catalase enzymes activity; TAS: total antioxidant status.

Evidence suggests modest improvements in symptoms such as attention and behavior with EPA-rich supplements, although effects vary between studies. Some trials showed reduction in symptoms (e.g., hyperactivity and stereotypic behaviors) following LC-PUFA supplementation, but findings are not uniform. These discrepancies may be attributed to variations in dosage, timing of intervention, baseline omega-3 status, genetic factors, and outcome assessment tools [51,52]. Evidence from RCTs indicates enhanced reading, spelling, and behavioral outcomes with supplemental omega-3s in affected children [70]. Although previous reviews have not consistently included the Oxford–Durham study within broader research on marine omega-3 for dyspraxia, marine long-chain omega-3s (EPA/DHA) from fish oil may improve reading, spelling and behavior in children with dyspraxia/DCD compared with placebo [70]. Only one major RCT in children with DCD aged 5–12 yrs has tested marine-derived fatty acids (fish oil and evening primrose oil). This study found improvements in reading, spelling and behavior, but not in motor coordination. Clinical evidence for marine lipid extracts like PCSO-524^®^ is limited to ADHD and other neurobehavioral studies and does not directly address DCD [77]. To date, no large RCTs evaluating motor outcomes have demonstrated significant benefits of marine omega-3s in children with DCD; most reported effects relate to behavioral or cognitive outcomes [76]. Furthermore, many studies focus on clinical symptoms rather than underlying mechanistic pathways (Figure 1) [78]. 

According to Chang et al., the dose for beneficial effects ranges in each condition as follows: (1) ADHD: a combination of EPA and DHA ≥ 750 mg/d, with a higher dose of EPA (up to 1200 mg/day) recommended for individuals with inflammation or allergic diseases, administered for 16−24 weeks; (2) MDD: a combination of EPA and DHA at 1000−2000 mg/day, with an EPA:DHA ratio of 2 to 1, for 12−16 weeks; and (3) ASD: a combination of EPA and DHA at 1300−1500 mg/day [74]. According to Strawn et al., the most effective dosage range for depression in children aged 6–17 years was 1290–4300 mg/day of combined EPA and DHA, resulting in a 50% reduction in symptoms, although most studies included in this analysis were of short duration; that is, they lasted for some weeks [75]. The proposed mechanisms underlying these effects are illustrated in Figure 2 [79], and the role of omega-3 fatty acids in cognitive development and neurological disorders during childhood in Figure 1 [78].

Omega-3 fatty acids play a role in activating the nuclear factor erythroid 2-related factor 2/antioxidant response element (Nrf2/ARE) pathway, protecting neurons from oxidative damage, regulating the gut–brain axis, and regulating the balance of gut microflora [79]. Nrf2 is a major regulator of the cellular antioxidant response and plays a central role in maintaining redox homeostasis [80]. Under basal conditions, Nrf2 is sequestered in the cytoplasm by Kelch-like ECH-associated protein 1 (Keap1) and targeted for proteasomal degradation; oxidative or electrophilic stress disrupts this interaction, allowing Nrf2 to translocate to the nucleus. There, Nrf2 binds to antioxidant response elements (AREs) in the promoters of target genes, inducing the transcription of a broad array of cytoprotective and antioxidant enzymes, including heme oxygenase-1 (HO-1), NAD(P)H:quinone oxidoreductase 1 (NQO1), superoxide dismutase (SOD), catalase (CAT), and glutathione system enzymes such as glutamate-cysteine ligase (GCLC) and glutathione synthetase (GSS), glutathione S-transferases (GSTs) and glutathione reductase (GSR) [80]. Through coordinated upregulation of these antioxidant and detoxifying enzymes, Nrf2 mitigates oxidative damage, limits inflammation, and enhances cellular resilience to stress [80].

Moreover, oxidative stress and inflammation play an important role in traumatic brain injury (TBI) and spinal cord injury (SCI), two conditions which are highly significant in children and represent a major public health concern [81]. The developing brain is particularly vulnerable to both primary injury and secondary injury mechanisms such as inflammation, excitotoxicity, and oxidative stress [82]. Because critical processes like synaptogenesis, myelination, and cortical maturation are still ongoing, TBI in childhood can disrupt normal neurodevelopment, leading to long-term cognitive, behavioral, emotional, and academic difficulties [83]. Pediatric SCI is particularly impactful because it occurs during a critical period of growth and neurodevelopment, leading to lifelong motor, sensory, autonomic, and psychosocial consequences [82]. A number of studies have reported that administration of omega-3 PUFAs exerted neuroprotection from TBI and SCI [81]. For example, DHA and ALA, a precursor of PUFAs, after being administered as a single intravenous bolus of 250 nmol/kg at 30 min after SCI, have been shown to exert neuroprotection against traumatic CNS. This protective effect was achieved through DHA-induced activation of Akt and c-AMP-response element-binding (CREB) protein [84]. In another study, a dietary supplement containing EPA and DHA at a 2:1 ratio, providing a dose of 24 mg/kg, was administered post-injury for 30 days [85]. The fatty acid dietary supplement protected from axonal damage and decreased the pro-apoptotic caspase-3 levels [85,86]. Caspase-3 is activated after mechanical-induced TBI, since injury leads to excitotoxic glutamate release, calcium overload, mitochondrial dysfunction, and excessive production of reactive oxygen species, all of which activate the mitochondrial apoptotic cascade [87].

Major depressive disorder (MDD) is also clinically significant in children [88]. Pediatric MDD can impair emotional, cognitive, social, and academic functioning and is associated with increased risk of substance use, anxiety disorders, and suicidality [88]. The administration of omega-3 PUFAs has been demonstrated to exhibit antidepressant activity through the modulation of inflammatory pathways and antioxidant mechanisms [89]. Moreover, some metabolites of omega-3 PUFAs seem to have an important role in their antidepressant ability [89,90]. In particular, Borsini et al., 2021 reported that the increase in metabolites from EPA and DHA in MDD patients was negatively correlated with depression severity [90]. These omega-3s’ antidepressant effects may be achieved through the modulation of the hypothalamic–pituitary–adrenal axis (HPA), the inhibition of neurodegeneration and the induction of neuronal plasticity [89]. Furthermore, DHA exerts potent neuroprotective effects in part through its enzymatic conversion to neuroprotectin D-1 (NPD-1), a bioactive lipid mediator generated in response to neural injury and oxidative stress [91,92]. NPD-1 modulates key survival pathways by suppressing pro-inflammatory signaling, inhibiting leukocyte infiltration, and downregulating pro-apoptotic proteins such as Bax while upregulating anti-apoptotic factors including Bcl-2 and Bcl-xL. In addition, NPD-1 limits oxidative damage and preserves mitochondrial integrity, thereby reducing caspase activation and neuronal apoptosis. Through these coordinated anti-inflammatory, anti-oxidative, and pro-survival actions, DHA-derived NPD-1 plays a critical role in maintaining neuronal homeostasis and promoting resilience in the injured or diseased brain [91,92].

### 2.2. Metabolic and Cardiometabolic Health

**Figure 3:**
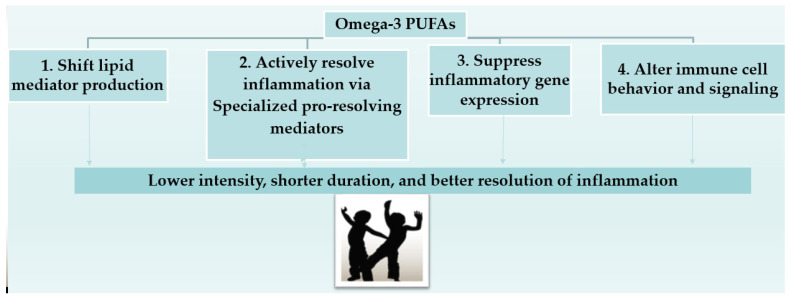
Omega-3 PUFAs and their anti-inflammatory properties [98].

LC-PUFAs also influence metabolic pathways critical for cardiometabolic health [60] as well as for lipid metabolism, insulin sensitivity, and inflammatory balance [93]. LC-PUFA supplementation can lower blood pressure [94] and triacylglycerol levels and may favorably modulate other lipid parameters (e.g., enhance fatty acid oxidation), adipocyte function and markers of metabolic syndrome, particularly in overweight or obese children, though effects on HDL cholesterol and blood glucose remain inconsistent and require further high-quality trials [55,56,57,58,59,93,95]. Systematic reviews in overweight and obese pediatric populations indicate that omega-3 intake may modestly reduce body mass index (BMI) [96], triacylglycerols, and insulin resistance (HOMA-IR), suggesting potential benefits for metabolic syndrome risk factors [97,98]. These findings are increasingly relevant given the global rise in childhood obesity and associated cardiometabolic risk [99]. Additionally, benefits reported include improved sleep [63], increased physical activity [62] and enhanced cognitive ability [61]. EPA and DHA exert anti-inflammatory effects by competing with arachidonic acid (AA) in eicosanoid pathways and increasing the production of pro-resolving mediators, thereby potentially reducing systemic inflammation. This mechanism may support metabolic and immune health in children [98]. The anti-inflammation effects of marine omega-3 PUFAs suggest that they may be useful as therapeutic agents in disorders with an inflammation component balance (Figure 3, adapted from Curioni et al. [98]). In children with inborn errors of metabolism (e.g., phenylketonuria), who are at risk of omega-3 deficiency and neurological impairment, supplementation has shown some promise in preserving neural function [100], but larger RCTs are necessary to establish clear therapeutic protocols (Table 3).

Omega-3 fatty acids, particularly EPA and DHA, reduce inflammatory responses through multiple complementary mechanisms: they activate PPARγ (peroxisome proliferator-activated receptor gamma), which increases anti-inflammatory gene expression and reduces the transcription of pro-inflammatory cytokines; they bind to GPR120 (G-protein coupled receptor 120) on macrophages and adipocytes, leading to the inhibition of inflammatory signaling cascades; they suppress the activation of MAPKs (mitogen-activated protein kinases), resulting in a reduction in the production of inflammatory mediators; and they inhibit the nuclear translocation of NF-κB (nuclear factor kappa-light-chain-enhancer of activated B cells), thereby reduce expression of pro-inflammatory genes such as TNF-α, IL-1β, and IL-6 [89,90]. Additionally, omega-3 fatty acids compete with arachidonic acid for PLA2 (phospholipase A2) and cyclooxygenase/lipoxygenase enzymes, leading to reduced synthesis of pro-inflammatory eicosanoids and increased production of specialized pro-resolving mediators (resolvins, protectins and maresins), which actively promote the resolution of inflammation [98].

After a 12-week intervention, LC-PUFA supplementation significantly decreased triacylglycerol, HOMA-IR, leptin, retinol-binding protein 4, selectin E and asymmetric dimethylarginine levels [55,56,57,58,59,93,95]. Moreover, LC-PUFA supplementation combined with lifestyle intervention displayed a significant reduction in triacylglycerol, asymmetric dimethylarginine and selectin E in comparison with lifestyle intervention alone [59]. Maternal DHA intake during pregnancy has also been shown to mitigate the association between childhood overweight condition or obesity and elevated blood pressure [94]. Juárez-López et al. at., in an open-label study (2013), found increased high-density lipoprotein cholesterol (HDL-C) and decreased low-density lipoprotein cholesterol (LDL-C) in obese and insulin-resistant children and adolescents receiving 1.8 g/day of LC-PUFAs for 12 weeks [93]. Agostoni et al. highlighted beneficial effects of omega-3 fatty acids on brain health during infancy [28], while improvements in behavior and learning with LC-PUFA supplementation have been observed in school-aged children [64]. Papamichael et al. published the first clinical trial evaluating a dietary source of omega-3 fatty acids in combination with a Mediterranean dietary pattern and found its potential use as adjunct therapy in the management of childhood asthma [65]. Fish, a key component of the Mediterranean diet, is a rich source of omega-3 PUFAs (EPA and DHA), which inhibit omega-6 PUFA (arachidonic acid) metabolism and interrupt inflammatory processes [65].

LC-PUFA supplementation (EPA and DHA) has been shown to modestly influence body mass index (BMI) and obesity-related parameters through multiple metabolic mechanisms [56]. Omega-3s activate PPARγ, which improves adipocyte differentiation and lipid metabolism, leading to increased fatty acid oxidation and decreased triacylglycerol accumulation. Through GPR120 activation, they enhance insulin sensitivity and attenuate chronic low-grade inflammation commonly associated with obesity. Omega-3 fatty acids also decrease the expression of lipogenic genes and adipocyte hypertrophy, while increasing mitochondrial β-oxidation in the liver and skeletal muscle. Additionally, their anti-inflammatory effects (via reduction in NF-κB and MAPK signaling) help reduce adipose tissue inflammation, a key contributor to obesity-related metabolic dysfunction. Clinically, supplementation is generally associated with modest reduction in BMI, waist circumference, and body fat mass, particularly when combined with caloric control and physical activity [59,101]. To examine the effect of LC-PUFAs in metabolic disorders, the findings of 12 RCTs included in this review are highlighted in Table 3 [28,55,56,57,58,59,60,61,62,63,64,65].

Western diets are typically characterized by omega-3/omega-6 ratios of approximately 1:15–20, which favors inflammation and obesity risk. Shifting the omega-3/omega-6 ratio to 1:4 or lower is associated with better metabolic outcomes in children. The benefits are more pronounced for triglyceride levels and markers of insulin resistance, with more modest effects on BMI and body fat [102]. LC-PUFAs, primarily EPA and DHA, have been widely studied for their potential cardiovascular benefits. Research indicates that regular consumption of these fatty acids, either through supplementation or fortified foods, can help reduce triacylglycerol levels, a known risk factor for cardiovascular disease. Additionally, omega-3s possess anti-inflammatory properties and may improve endothelial function, supporting vascular health [103]. Clinical studies have also suggested modest reductions in blood pressure and decreased platelet aggregation, both of which contribute to lowering the risk of heart attacks and strokes [104]. EPA and DHA, the two primary LC-PUFAs, work synergistically to support cardiovascular health, with EPA particularly effective at lowering triglycerides and DHA contributing to improved heart rhythm and blood pressure regulation. Clinical evidence suggests that a combined intake of EPA and DHA offers broader systemic benefits than either alone, enhancing lipid metabolism, supporting vascular integrity, and modulating inflammatory pathways. Therefore, while both are critical for heart health, their complementary effects underline the importance of considering full-spectrum omega-3 supplementation rather than focusing solely on EPA [97,105].

### 2.3. Omega-3-Fortified Foods and General Health

Omega-3-enhanced foods such as soups, pasta and spreads enriched with microalgae provide a practical dietary approach to increase intake without supplementation [106]. Although enriched foods may offer slightly less pronounced benefits than supplements, studies suggest comparable improvements in lipid profiles and cardiovascular risk markers. However, some other food products such as spreads may be high in trans fats and may not confer beneficial effects. Overall, consistent omega-3 intake through either supplements or fortified foods appears to offer protective effects for the cardiovascular system, though optimal dosage and long-term outcomes remain under investigation [105]. Marine omega-3 supplements (fish oil, cod liver oil, and algal oil) provide concentrated EPA and DHA and are widely used to increase intake beyond what diet alone typically delivers. Algal oil is particularly relevant for vegetarian or allergen-sensitive populations and has been shown to effectively raise DHA levels in children [107]. RCTs examining supplementation of various dosages and formulations showed that doses exceeding 300–500 mg/day of combined EPA and DHA may be necessary to achieve measurable cognitive and behavioral effects [25,29,30,31]. Overall, supplementation appears to be most beneficial in children with low baseline omega-3 status or specific developmental or metabolic risk factors [60]. Moreover, supplements are generally well tolerated in pediatric populations. Rarely, high doses may affect coagulation, underscoring the need for appropriate dosing and medical supervision [108]. Mild gastrointestinal symptoms are the most commonly reported side effects, while concerns regarding bleeding risk are minimal at typical pediatric doses [109]. According to the EFSA, omega-3 fatty acids do not cause any side effects at intakes up to 5–6 g/day, provided that an adequate proportion of omega-3 PUFAs (1–2% of daily energy intake) is maintained [110,111]. EFSA has also proposed a labeling reference intake of 2 g/day for ALA [112]. Although environmental contaminants such as mercury in certain fish species have raised concerns, current dietary guidelines emphasize that the benefits of consuming low-mercury, omega-3-rich fish outweigh potential risks [113,114].

**Figure 4:**
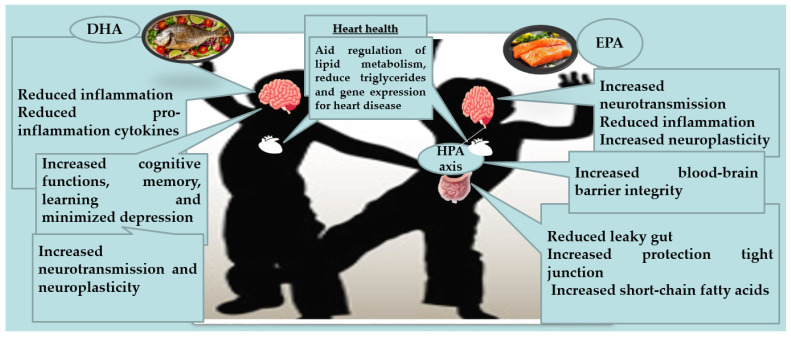
Health benefits of LC-PUFAs consumption during childhood (HPA axis: hypothalamic–pituitary–adrenal axis) [15,66,118].

Fortified foods (e.g., omega-3-enriched milk, egg and spreads) and enhanced infant formulas, especially for preterm infants [15], provide additional dietary sources of DHA and EPA. These products have demonstrated improvements in visual and neural indices in infants [25,26,27] and may help meet dietary recommendations, especially in populations including children with low fish intake [28,35]. However, efficacy depends on product formulation and dosage [115,116]. Natural dietary sources such as fatty fish and seafood remain foundational for achieving adequate omega-3 status. Many studies document low intake of EPA and DHA among children, often below recommended levels, which may contribute to suboptimal developmental and metabolic outcomes [117]. However, further high-quality evidence is needed to establish optimal dosing, timing, and delivery methods [15,66,118]. Beyond neurodevelopment and metabolism, marine omega-3 fatty acids support broader aspects of health [119]. Fish consumption is associated with lower risk of heart disease and stroke in adults, and early dietary patterns may influence lifelong cardiovascular risk, though pediatric data are limited [120]. Omega-3s also modulate immune responses, with some evidence suggesting reduced duration or severity of infections and inflammation. Adequate omega-3 intake contributes to healthy growth trajectories, though its effects are subtler compared with those observed in specific clinical conditions [15,118]. Beyond neurodevelopment and metabolism, omega-3 fatty acids also contribute to immune modulation (Table 4) [28,35] and the resolution of inflammation [66], potentially reducing the severity or duration of infections in children (Figure 4) [15,16,118].

Regular consumption of fish and marine omega-3s also provides additional nutrients such as high-quality protein, iodine, selenium, and vitamin D, which collectively support overall growth and health [112]. As reported above, marine omega-3 supplements are generally safe for children when used appropriately [119]. Dietary recommendations vary by age but often aim to ensure sufficient EPA and DHA intake through, when necessary, a combination of diet and supplements, according to the International Society for the Study of Fatty Acids and Lipids (ISSFAL) [121,122]. Public health agencies (e.g., EFSA, FAO, and WHO) typically recommend around 100–250 mg/day of EPA and DHA for children, with higher intakes for specific clinical conditions or therapeutic goals (Table 5) [118,122]. Some reviews suggest that ≥450 mg/day of combined DHA and EPA may benefit cognitive outcomes [121]. Overall, safety profiles are generally favorable, but concerns about contaminants (e.g., mercury and PCBs) found in fish and potential bleeding risk at high doses require consideration [123].

Summarizing, the review outcomes are as follows: (1) Strongest evidence: visual development, ADHD symptom reduction, and triacylglycerol lowering. (2) Moderate evidence: cognitive attention benefits and metabolic risk reduction. (3) Mixed evidence: autism core symptoms and long-term IQ improvement. (4) Best responders: children with low baseline omega-3 intake.

## 3. Methods

In order to search for the information, a literature search was carried out in the PubMed, Web of Science, Scopus and Cochrane Library databases up to January 2026. The search also included articles which were bibliographic references to the articles that were studied. This study has inclusion criteria involving epidemiology data (all age groups from infancy to childhood and adulthood, ethnicities, and socio-economic status) and the design of the studies (controlled trials, cohort studies, cross-sectional studies, and systematic reviews, but also with an emphasis on randomization, variability of the used questionnaires, and sample size), whereas exclusion criteria were studies with limited sizes and concerns about risk of bias.

This review was performed according to the Preferred Reporting Items for Systematic Reviews and Meta-Analyses (PRISMA) 2020 guidelines and checklist [124]. Moreover, randomized clinical trials were included to assess the dose-dependent effects of omega-3 fatty acids on metabolic and neurological disorders as well as overall health [125]. Additionally, this review focused on evaluating the impact of LC-PUFAs on general health.

Regarding the methodology of this narrative review, a comprehensive search encompassing three databases was conducted as follows: (1) The search on PubMed and Scopus involved the utilization of MeSH terms specifically focusing on “#Health,” AND/OR “#Diet,” AND/OR “#Polyunsaturated fatty acids,” AND/OR “#Intervention’’, “#Randomized clinical trial,” AND/OR “#Children,” AND/OR “#Adolescents,” AND/OR #Development,” AND/OR “#Cognitive function,” AND/OR “#Neurological disorders”, AND/OR “#Physical Function”, “#Metabolic disorders,” AND/OR “#Inflammation,” AND/OR “#Marine sources,” AND/OR “#Supplements,” AND/OR “#Enhanced foods’ “. The aim was to investigate the relationship between the dose of omega-3 PUFAs and their impact on overall health, but also to shed light on the complex molecular pathways that support their therapeutic role and emphasize how to incorporate foods that contain omega-3 fats into children’s meals to reap their numerous health benefits. The preliminary search produced 202 articles, with 57 duplicates excluded, resulting in 145 articles for further analysis. (2) The search strategy based on the Web of Science database concentrated on identifying articles related to “#Health,” AND/OR “#Diet,” AND/OR “#Omega-3 polyunsaturated fatty acids,” AND/OR “#Intervention’’, AND/OR “#Randomized clinical trial,” AND/OR “#Children,” AND/OR “#Adolescents,” AND/OR “#Development,” AND/OR “#Cognitive function,” AND/OR “#Neurological disorders”, AND/OR “#Metabolic disorders”, AND/OR “#Strength,” AND/OR “#Inflammation,” AND/OR “#Mental health,” AND/OR “#Supplements,” AND/OR “#Enhanced foods,” AND/OR “#Cardiovascular disease,” AND/OR “#Cancer”, AND/OR “#Marine sources,” AND/OR “#Nutritional profile,” AND/OR “#Microalgae”. Additional terms such as “#Food” AND/OR “#Nutrition” were included to broaden the search. The objective was to explore dietary habits, nutritional interventions and the role of various diet patterns in modulating specific bioactive compounds and addressing related disorders. The initial search yielded 5 articles, of which 3 duplicates were removed, leaving 2 articles for detailed scrutiny. (3) The search using the Cochrane Library emphasized “#Omega-3 polyunsaturated fatty acids”, AND/OR “#Interventions”, AND/OR “#Nutrition”, AND/OR “#Overall health”, AND/OR “#Randomized clinical trials”, with an additional keyword “#Supplementation” AND/OR “#Enhanced foods”. The primary aim was to identify studies examining the association between omega-3 PUFA consumption and overall and mental health, with particular attention to guidance provided by health professionals (Table 6). Inclusion and exclusion criteria were highlighted in Table 7.

## 4. Conclusions

Marine LC-PUFAs, especially DHA and EPA, are critical structural and functional components of the developing brain and retina, participating in key processes such as neuronal growth, synapse formation, and neurotransmission. Evidence consistently suggests that adequate omega-3 status during pregnancy, infancy, and early childhood supports optimal neurodevelopment, although effects in healthy populations are often modest and occasionally inconsistent across studies. Some recent reviews report that higher DHA/EPA levels during prenatal and early postnatal periods are associated with modest improvements in specific cognitive and communication outcomes, though not uniformly across all measures of general cognitive development. In clinical subgroups such as children with neurodevelopmental disorders (e.g., ADHD and ASD), some randomized controlled trials and umbrella reviews show reductions in hyperactivity or repetitive behaviors and occasional gains in attention or memory with omega-3 supplementation, particularly at higher doses (e.g., EPA ≥500 mg/d), although overall results vary and effect sizes tend to be small. Additionally, emerging evidence suggests that omega-3 PUFAs may modulate immune responses and inflammation, with some indication of reduced respiratory infections or inflammatory markers, and potentially beneficial effects on motor coordination and information processing in select populations, but high-quality data remain limited.

In terms of metabolic and broader health outcomes, the present review indicates that omega-3 supplementation alone does not consistently influence growth patterns (weight and length) or long-term cognitive development in all children, nor does it reliably prevent disorders such as obesity or asthma when used in isolation. Some evidence supports the role of omega-3s in metabolic regulation and cell membrane function, which could have implications for future cardiometabolic health if healthy eating patterns—including regular fatty fish consumption—are established early. The strongest and most robust findings suggest benefits of marine omega-3 intake in improving specific aspects of behavior and neurocognitive function in at-risk pediatric groups, as well as a safe profile in children when administered at appropriate doses. These observations underscore omega-3 status as a modifiable nutritional factor in child health. Continued well-designed, long-term trials and standardized outcome measures are urgently needed to clarify dose–response relationships, identify population subgroups most likely to benefit, and establish clear clinical guidelines.

## Figures and Tables

**Table 3:** Effects of marine omega-3 fatty acids on metabolic health in children according to randomized clinical trials.

Study (Year)	Age Group	Participants (*n*)	Population	Dose	Duration	Main Outcomes	Key Findings	References
Lopez et al. (2019)	8–16 years	*n* = 366	Overweight/Obese children	1200 mg/day PUFAs	3 months	HOMA, BMI, insulin resistance	Changes in weight, insulin, and HOMA were not related with supplementation	[55]
Rosas-Nexticapa et al. (2017)	10–12 years	*n* = 121	Overweight/Obese children	2 or 3 gummies (70 or 105 mg DHA) and 10 or 15 g salmon per day	3 months	Weight, height, BMI, waist-hip ratio, and serum parameters such as glucose, triacylglycerol, cholesterol, HDL-C, VLDL and LDL	Beneficial effects on dyslipidemia and, potentially reducing the risk of developing cardiovascular diseases	[56]
García-Cervera et al. (2015)	10–12 years	*n* = 303	Obese children	Fish (73.2–97.6 mg omega-3 fatty acids) or gummies (210–350 mg, omega-3 fatty acids)	1 month	Triacylglycerol, total cholesterol and body mass index	Dietary intake of gummies enriched with omega-3 fatty acids induced greater changes in triacylglycerol’s levels, total cholesterol and body mass index compared with dietary fish intake	[57]
Del-Río-Navarro et al. (2019)	10–16 years	*n* = 130	Pediatric patients with obesity and hypertriglyceridemia	3 g/day omega-3 fatty acids	12 weeks	Triacylglycerol concentrations	Triacylglycerol concentrations decreased by 39.1% in the omega-3 group and 14.6% in the placebo group	[58]
Huang et al. (2019)	10–16 years	*n* = 69	Overweight/Obese children	3 g/day PUFA supplementation (2000 mg EPA and 1000 mg DHA) (ratio EPA: DHA 2:1)	12 weeks	Triacylglycerol, HOMA, leptin, RBP4, ADMA and sE	Significant improvement in dyslipidemia, insulin resistance, adipokine abnormality, and endothelial dysfunction	[59]
García-López et al. (2016)	11–12 years	*n* = 69	Children with metabolic syndrome	2.4 g/day DHA	1 month	Lipid profile, fasting glucose levels, and blood pressure	Improved triacylglycerol; minimal changes in HDL/LDL; reduced fasting glucose levels and blood pressure	[60]
Agostoni et al. (2006)	Infants (entry) and 1 year of age	*n* = 42	Phenylketonuria (PKU)	DHA-enriched supplements	Long-term	Cognitive and neural function	Prevention of DHA deficiency; neuroprotective effects	[28]
Sittiprapapon et al. (2022)	6–12 years	*n* = 124	Healthy children	Low-dose fish oil (260 mg DHA); high-dose fish oil (520 mg DHA)	12 weeks	Cognition	Generally neutral effects unless baseline deficiency was present	[61]
Vuholm et al. (2021)	8–9 years	*n* = 199	Healthy children	~300 g/week oily fish	12 weeks	Sleep and physical activity	Oily fish intake altered sleep and physical activity patterns	[62]
Montgomery et al. (2014)	7–9 years	*n* = 395	Healthy children	Algal DHA supplementation (600 mg/day)	16 weeks	Improved sleep	Higher blood levels of DHA possibly related to better sleep in children	[63]
Richardson et al. (2012)	7–9 years	*n* = 74	Healthy children	600 mg/day DHA (from algal oil)	16 weeks	Improvement in child behavior and learning	Parent-rated behavior problems (ADHD-type symptoms) significantly reduced by active treatment	[64]
Papamichael et al. (2018)	5–12 years	*n* = 64	Children with asthma	50 g cooked fish per week	6 months	Asthma control, lipids, and quality of life	Dietary source of omega-3 fatty acids in combination with a Mediterranean dietary pattern may be used as adjunct therapy	[65]

PUFAs: polyunsaturated fatty acids; RBP4: retinol-binding protein 4; sE: selectin E; ADMA: asymmetric dimethylarginine; HDL-C: high-density lipoprotein cholesterol; LDL-C: low-density lipoprotein cholesterol; VLDL-C: very-low-density lipoprotein cholesterol.

**Table 4:** Omega-3 fortified foods and general health outcomes in children.

Food Type	Age Group	Omega-3	Duration	Main Outcomes	Key Findings	References
DHA-fortified infant formula	Infants	0.2–0.35% DHA	6–12 months	Visual and neural development	Consistent visual benefits	[25,26,27]
Omega-3 enriched milk/eggs	School-aged children	100–250 mg/day	3–6 months	Omega-3 status and cognition	Improved blood DHA levels; limited functional effects	[2]
Fish consumption (dietary)	Children and adolescents	1–2 servings/ week	Long-term	Growth and immunity	Supports overall health and nutrition quality	[28,35]
Algal DHA foods	Vegetarian children	100–300 mg/day	Variable	DHA status	Effective alternative to fish sources	[115,116]

**Table 5:** Recommended intake ranges of marine omega-3 fatty acids in children [118,122].

Age Group	Recommended Intake (EPA + DHA)	Source
Infants (0–12 months)	100 mg DHA/day	WHO/FAO
Infants (0–6 months)	200 mg/day DHA/day	ISSFAL
Infants (6–24 months)	100–120 mg DHA/day	ISSFAL
Toddlers (1–3 years)	100–150 mg/day	EFSA
Children (2–4 years)	100–150 mg/day	ISSFAL
Children (4–8 years)	150–200 mg/day	EFSA
Children (4–6 years)	50–200 mg/day	ISSFAL
Children (9–13 years)	200–250 mg/day	EFSA
Children (6–10 years)	200–250 mg/day	ISSFAL
Adolescents (14–18 years)	250–500 mg/day	EFSA/AHA
Adolescents (14–18 years)	250–500 mg/day	ISSFAL
Therapeutic doses (ADHD; metabolic risk)	500–1000 mg/day	Clinical trials

**Table 6:** Details of the search process and the unique contributions of each database to this study.

Database	Keywords	MeSH Terms (PubMed)	Initial Articles	Duplicates Removed	Final Articles for Analysis	Contribution to Study	Reason for Inclusion
PubMed	#Health, #Diet, #Omega-3 Polyunsaturated fatty acids, #Intervention, #Randomized clinical trial, #Children, #Adolescent, #Development, #Cognitive function, #Metabolic disorders, #Neurological disorders, #Physical function, #Inflammation. #Supplements, #Enhanced foods, and #Marine sources	#Omega-3 Poly-unsaturated fatty acids, #,”Intervention, #Randomized clinical trial, #Older adults, #Dairy Cow, #Nutritional profile, and #Milk	190	48	142	Provided a broad understanding of the interplay between diet, food consumption, dietary interventions, and mental health benefits; MeSH terms ensured precision in the search for relevant studies	Widely recognized as a premier biomedical database, frequently used for reviews in healthcare research
Web of Science	#Health, #Diet, #Omega-3 polyunsaturated fatty acids, #Intervention, #Randomized clinical trial, #Children, #Adolescent, #Development #Cognitive function, #Metabolic disorders, #Neurological disorders, #Strength, and #Inflammation, #Mental health, #Cardiovascular disease, #Cancer, #Food, #Nutrition, #Marine sources, #Supplementation, and #Enhanced foods	N/A (Web of Science does not use MeSH terms)	5	3	2	Enhanced the overall coverage of literature related to dietary interventions, and their impact on mental health	Provides a multidisciplinary approach, covering a wide range of scientific disciplines
Scopus	#Health, #Diet, #Omega-3 Poly-unsaturated fatty acids, #Intervention, #Randomized clinical trial, #Children, #Adolescent, #Development, #Cognitive function, #Supplements, #Enhanced foods, #Strength, and #Inflammation	#Omega-3 Poly-unsaturated fatty acids, #Randomized clinical trial, #Health	12	9	3	Strengthened the evidence base by focusing on dietary interventions, and their impact on mental health; MeSH terms ensured specificity in selecting relevant studies	Renowned for reviews and emphasizing evidence-based interventions in healthcare research
Cochrane Library	#Omega-3 polyunsaturated fatty acids, #Interventions, #Nutrition, #Overall health, #Randomized clinical trials, #Supplementation and #Enhanced foods	#Omega-3 polyunsaturated fatty acids, #Interventions, #Health	30	28	2	Strengthened the evidence base by focusing on bioactive compounds in meals and snacks related to evidence-based interventions; MeSH terms ensured specificity in selecting relevant studies	Renowned for reviews and emphasizing evidence-based interventions in healthcare research

**Table 7:** Inclusion and exclusion criteria.

Inclusion Criteria	Exclusion Criteria
Published in English	Case reports and practical guidelines
Randomized controlled trials or reviews	Sample parameters (small sample)
Participants aged <18 years old	No comparator group (i.e., control or alternative dietary intervention)
Studies with a minimum 3 month follow-up and a minimum of 24 participants	Does not report primary and/or secondary outcomes
Meta-analyses and reviews	

## Data Availability

No new data were created or analyzed in this study. Data sharing is not applicable to this article.

## References

[1] Bhutta Z.A., Guerrant R.L., Nelson C.A. (2017). Neurodevelopment, nutrition, and inflammation: The evolving global child health landscape. Pediatrics.

[2] Uauy R., Dangour A.D. (2009). Fat and fatty acid requirements and recommendations for infants of 0–2 years and children of 2–18 years. Ann. Nutr. Metab..

[3] Simopoulos A.P. (2020). Omega-3 Fatty Acids in Growth and Development. Omega-3 Fatty Acids in Health and Disease.

[4] Saidaiah P., Banu Z., Khan A.A., Geetha A., Somraj B. (2024). A comprehensive review of Omega-3 fatty acids: Sources, industrial applications, and health benefits. Ann. Phytomed..

[5] Innis S.M. (2007). Dietary (n-3) fatty acids and brain development1. J. Νutr..

[6] Georgieff M.K., Ramel S.E., Cusick S.E. (2018). Nutritional influences on brain development. Acta Paediatr..

[7] Parks C.A., Brett N.R., Agellon S., Lavery P., Vanstone C.A., Maguire J.L., Rauch F., Weiler H.A. (2017). DHA and EPA in red blood cell membranes are associated with dietary intakes of omega-3-rich fish in healthy children. Prostaglandins Leukot. Essent. Fat. Acids.

[8] Ahmad M.F., Alsayegh A.A., Khanam A., Ahmed A., Raposo A., Bantun F., Zeyaullah M., Babalghith A.O., Aldairi A.F., Mozaffar B. (2025). Efficacy of omega-3 fatty acids as a functional food: A multifaceted approach to health reinforcement. J. Sci. Food Agric..

[9] Cholewski M., Tomczykowa M., Tomczyk M. (2018). A comprehensive review of chemistry, sources and bioavailability of omega-3 fatty acids. Nutrients.

[10] Fan L., Wang X., Szeto I.M.-Y., Liu B., Sinclair A.J., Li D. (2024). Dietary intake of different ratios of ARA/DHA in early stages and its impact on infant development. Food Funct..

[11] Wei W., Cheng Z., Wang X., Chang M., Xie D., Sun C., Guo X., Li B. (2025). The potential of marine oils as functional foods: Nutritional composition, health benefits, applications, and future perspectives. Food Rev. Int..

[12] Ahmmed M.K., Ahmmed F., Tian H., Carne A., Bekhit A.E.D. (2020). Marine omega-3 (n-3) phospholipids: A comprehensive review of their properties, sources, bioavailability, and relation to brain health. Compr. Rev. Food Sci. Food Saf..

[13] Panse M.L., Phalke S.D. (2019). Omega-3 Beverages. Value-Added Ingredients and Enrichments of Beverages.

[14] Mirehsanpazir S., Gharachorloo M., Asadi G. (2021). Preparation and formulation of functional half-fat spread butter with micro-emulsions containing omega-3. Iran. Food Sci. Techn Res. J..

[15] Koletzko B., Bergmann K., Brenna J.T., Calder P.C., Campoy C., Clandinin M.T., Colombo J., Daly M., Decsi T., Demmelmair H. (2020). Should formula for infants provide arachidonic acid along with DHA? A position paper of the European Academy of Paediatrics and the Child Health Foundation. Am. J. Clin. Nutr..

[16] Ruiz-Lopez N., Stubhaug I., Ipharraguerre I., Rimbach G., Menoyo D. (2015). Positional distribution of fatty acids in triacylglycerols and phospholipids from fillets of Atlantic salmon (*Salmo salar*) fed vegetable and fish oil blends. Mar. Drugs.

[17] Christie W.W., Harwood J.L. (2020). Oxidation of polyunsaturated fatty acids to produce lipid mediators. Essays Biochem..

[18] Gupta J., Gupta R. (2020). Nutraceutical status and scientific strategies for enhancing production of omega-3 fatty acids from microalgae and their role in healthcare. Curr. Pharm. Biotechnol..

[19] Bauer E., Jakob S., Mosenthin R. (2005). Principles of physiology of lipid digestion. Asian-Aust. J. Anim. Sci..

[20] Bailey E., Wojcik J., Rahn M., Roos F., Spooren A., Koshibu K. (2025). Comparative Bioavailability of DHA and EPA from Microalgal and Fish Oil in Adults. Int. J. Mol. Sci..

[21] Makay K., Griehl C., Schilling S., Grewe C. (2025). Omega-3 Source Matters: Comparative Lipid Signatures and Quantitative Distribution of EPA/DHA Across Marine Resources. Mar. Drugs.

[22] Arterburn L.M., Hall E.B., Oken H. (2006). Distribution, interconversion, and dose response of n − 3 fatty acids in humans. Am. J. Clin. Nutr..

[23] Nesheim M.C., Oria M., Yih P.T., Committee on a Framework for Assessing the Health, Environmental, and Social Effects of the Food System, Food and Nutrition Board, Board on Agriculture and Natural Resources, Institute of Medicine, National Research Council (2015). Dietary recommendations for fish consumption. A Framework for Assessing Effects of the Food System.

[24] Sherzai D., Moness R., Sherzai S., Sherzai A. (2023). A Systematic Review of Omega-3 Fatty Acid Consumption and Cognitive Outcomes in Neurodevelopment. Am. J. Lifest Med..

[25] Qawasmi A., Landeros-Weisenberger A., Leckman J.F., Bloch M.H. (2012). Meta-analysis of long-chain polyunsaturated fatty acid supplementation of formula and infant cognition. Pediatrics.

[26] von Schacky C. (2020). Omega-3 index in 2018/19. Proc. Nutr. Soc..

[27] Lapillonne A., Jensen C.L. (2009). Reevaluation of the DHA requirement for the premature infant. Prostaglandins Leukot. Essent. Fat. Acids.

[28] Agostoni C., Harvie A., McCulloch D.L., Demellweek C., Cockburn F., Giovannini M., Murray G., Harkness R.A., Riva E. (2006). A randomized trial of long-chain polyunsaturated fatty acid supplementation in infants with phenylketonuria. Dev. Μed. Child. Neurol..

[29] Gould J.F., Anderson P.J., Yelland L.N., Gibson R.A., Makrides M. (2021). The influence of prenatal dha supplementation on individual domains of behavioral functioning in school-aged children: Follow-up of a randomized controlled trial. Nutrients.

[30] Birch E.E., Carlson S.E., Hoffman D.R., Fitzgerald-Gustafson K.M., Fu V.L., Drover J.R., Castañeda Y.S., Minns L., Wheaton D.K., Mundy D. (2010). The DIAMOND (DHA Intake and Measurement of Neural Development) Study: A double-masked, randomized controlled clinical trial of the maturation of infant visual acuity as a function of the dietary level of docosahexaenoic acid123. Am. J. Clin. Nutr..

[31] Escolano-Margarit M.V., Ramos R., Beyer J., Csábi G., Parrilla-Roure M., Cruz F., Perez-Garcia M., Hadders-Algra M., Gil A., Decsi T. (2011). Prenatal DHA Status and Neurological Outcome in Children at Age 5.5 Years Are Positively Associated^1234^. J. Nutr..

[32] von Schacky C. (2021). Importance of EPA and DHA blood levels in brain structure and function. Nutrients.

[33] Cooper R.E., Tye C., Kuntsi J., Vassos E., Asherson P. (2015). Omega-3 polyunsaturated fatty acid supplementation and cognition: A systematic review and meta-analysis. J. Psychopharmacol..

[34] Hawkey E., Nigg J.T. (2014). Omega−3 fatty acid and adhd: Blood level analysis and meta-analytic extension of supplementation trials. Clin. Psychol. Rev..

[35] Meyer B.J. (2016). Australians are not meeting the recommended intakes for omega-3 long chain polyunsaturated fatty acids: Results of an analysis from the 2011–2012 national nutrition and physical activity survey. Nutrients.

[36] Gould J.F., Treyvaud K., Yelland L.N., Anderson P.J., Smithers L.G., Gibson R.A., McPhee A.J., Makrides M. (2016). Does n-3 LCPUFA supplementation during pregnancy increase the IQ of children at school age? Follow-up of a randomised controlled trial. BMJ Open.

[37] Willatts P., Forsyth S., Agostoni C., Casaer P., Riva E., Boehm G. (2013). Effects of long-chain PUFA supplementation in infant formula on cognitive function in later childhood. Am. J. Clin. Nutr..

[38] San Mauro Martin I., Sanz Rojo S., González Cosano L., Conty de la Campa R., Garicano Vilar E., Blumenfeld Olivares J.A. (2022). Impulsiveness in children with attention-deficit/hyperactivity disorder after an 8-week intervention with the Mediterranean diet and/or omega-3 fatty acids: A randomised clinical trial. Neurologia.

[39] Carucci S., Romaniello R., Demuru G., Curatolo P., Grelloni C., Masi G., Liboni F., Mereu A., Contu P., Lamberti M. (2022). Omega-3/6 supplementation for mild to moderate inattentive ADHD: A randomised, double-blind, placebo-controlled efficacy study in Italian children. Eur. Arch. Psychiatry Clin. Neurosci..

[40] Barragán E., Breuer D., Döpfner M. (2017). Efficacy and safety of omega-3/6 fatty acids, methylphenidate, and a combined treatment in children with ADHD. J. Atten. Disord..

[41] Assareh M., Davari Ashtiani R., Khademi M., Jazayeri S., Rai A., Nikoo M. (2017). Efficacy of Polyunsaturated Fatty Acids (PUFA) in the Treatment of Attention Deficit Hyperactivity Disorder: A Randomized, Double-Blind, Placebo-Controlled Clinical Trial. J. Atten. Disord..

[42] Bos D.J., Oranje B., Veerhoek E.S., Van Diepen R.M., Weusten J.M.H., Demmelmair H., Koletzko B., de Sain-van der Velden M.G.M., Eilander A., Hoeksma M. (2015). Reduced Symptoms of Inattention after Dietary Omega-3 Fatty Acid Supplementation in Boys with and without Attention Deficit/Hyperactivity Disorder. Neuropsychopharmacology.

[43] Berger G., Häberling I., Emery S., Albermann M., Baumgartner N., Nalani K., Strumberger M., Wöckel L., Erb S., Bachmann S. (2026). ω-3 Fatty Acids in Pediatric Major Depressive Disorder: A Randomized Clinical Trial. JAMA Netw. Open.

[44] Gabbay V., Freed R.D., Alonso C.M., Senger S., Stadterman J., Davison B.A., Klein R.G. (2018). A double-blind placebo-controlled trial of omega-3 fatty acids as a monotherapy for adolescent depression. J. Clin. Psychiatry.

[45] Häberling I., Berger G., Schmeck K., Held U., Walitza S. (2019). Omega-3 Fatty Acids as a Treatment for Pediatric Depression. A Phase III, 36 Weeks, Multi-Center, Double-Blind, Placebo-Controlled Randomized Superiority Study. Front. Psychiatry.

[46] Fristad M.A., Young A.S., Vesco A.T., Nader E.S., Healy K.Z., Gardner W., Wolfson H.L., Arnold L.E. (2015). A Randomized Controlled Trial of Individual Family Psychoeducational Psychotherapy and Omega-3 Fatty Acids in Youth with Subsyndromal Bipolar Disorder. J. Child. Adolesc. Psychopharmacol..

[47] Katrenčíková B., Vaváková M., Paduchová Z., Nagyová Z., Garaiova I., Muchová J., Ďuračková Z., Trebatická J. (2021). Oxidative Stress Markers and Antioxidant Enzymes in Children and Adolescents with Depressive Disorder and Impact of Omega-3 Fatty Acids in Randomised Clinical Trial. Antioxidants.

[48] Mazahery H., Conlon C.A., Beck K.L., Mugridge O., Kruger M.C., Stonehouse W., Camargo C.A., Meyer B.J., Tsang B., Jones B. (2019). A randomised controlled trial of vitamin D and omega-3 long chain polyunsaturated fatty acids in the treatment of irritability and hyperactivity among children with autism spectrum disorder. J. Autism Dev. Disord..

[49] Beblo S., Reinhardt H., Demmelmair H., Muntau A.C., Koletzko B. (2007). Effect of fish oil supplementation on fatty acid status, coordination, and fine motor skills in children with phenylketonuria. J. Pediatr..

[50] Nemets H., Nemets B., Apter A., Bracha Z., Belmaker R. (2006). Omega-3 treatment of childhood depression: A controlled, double-blind pilot study. Am. J. Psychiatry.

[51] Fristad M.A., Vesco A.T., Young A.S., Healy K.Z., Nader E.S., Gardner W., Seidenfeld A.M., Wolfson H.L., Arnold L.E. (2019). Pilot randomized controlled trial of omega-3 and individual–family psychoeducational psychotherapy for children and adolescents with depression. J. Clin. Child Adolesc. Psychol..

[52] Wang J., Li S., Wang D., Gao Y., Wang Q., Wang T., Wang G., Peng D., Qiao Y., Zhou J. (2025). Effects of Omega-3 PUFAs on lipid profiles and antioxidant response in depressed adolescents: A metabolomic and lipidomic study. Redox Biol..

[53] Widenhorn-Müller K., Schwanda S., Scholz E., Spitzer M., Bode H. (2014). Effect of supplementation with long-chain ω-3 polyunsaturated fatty acids on behavior and cognition in children with attention deficit/hyperactivity disorder (ADHD): A randomized placebo-controlled intervention trial. Prostaglandins Leukot. Essent. Fat. Acids.

[54] Parellada M., Llorente C., Calvo R., Gutierrez S., Lázaro L., Graell M., Guisasola M., Dorado M.L., Boada L., Romo J. (2017). Randomized trial of omega-3 for autism spectrum disorders: Effect on cell membrane composition and behavior. Eur. Neuropsychopharmacol..

[55] López-Alarcón M., Inda-Icaza P., Márquez-Maldonado M., Armenta-Álvarez A., Barbosa-Cortés L., Maldonado-Hernández J., Piña-Aguero M., Barradas-Vázquez A., Núñez-García B., Rodríguez-Cruz M. (2019). A randomized control trial of the impact of LCPUFA-ω3 supplementation on body weight and insulin resistance in pubertal children with obesity. Pediatr. Obes..

[56] Rosas-Nexticapa M., Caballero-Rodríguez D.A., Herrera-Meza S., Acosta-Mesa H.-G., Santiago-Roque I., Figueroa-Valverde L., García-Cervera E., Pool-Gómez E., Cervantes-Ortega C., Mateu-Armand V. (2017). Supplementation effect of omega-3 fatty acids in overweight and obese Mexican schoolchildren. Interciencia.

[57] García-Cervera E., Figueroa-Valverde L., Gómez E.P., Díaz-Cedillo F., Rosas-Nexticapa M., Sarabia-Alcocer B., Nájera-Medina O., Villanueva R., García-López S. (2015). Effect of omega-3 fatty acids on triglycerides and BMI levels in obese children. Curr. Pediatr. Res..

[58] Del-Río-Navarro B.E., Miranda-Lora A.L., Huang F., Hall-Mondragon M.S., Leija-Martínez J.J. (2019). Effect of supplementation with omega-3 fatty acids on hypertriglyceridemia in pediatric patients with obesity. J. Pediatr. Endocrinol. Metab..

[59] Huang F., del-Río-Navarro B.E., Leija-Martinez J., Torres-Alcantara S., Ruiz-Bedolla E., Hernández-Cadena L., Barraza-Villarreal A., Romero-Nava R., Sanchéz-Muñoz F., Villafaña S. (2019). Effect of omega-3 fatty acids supplementation combined with lifestyle intervention on adipokines and biomarkers of endothelial dysfunction in obese adolescents with hypertriglyceridemia. J. Nutr. Bio.

[60] García-López S., Arriaga R.E.V., Medina O.N., López C.P.R., Figueroa-Valverde L., Cervera E.G., Skidmore O.M., Rosas-Nexticapa M. (2016). One month of omega-3 fatty acid supplementation improves lipid profiles, glucose levels and blood pressure in overweight schoolchildren with metabolic syndrome. J. Pediatr. Endocrinol. Metab..

[61] Sittiprapaporn P., Bumrungpert A., Suyajai P., Stough C. (2022). Effectiveness of Fish Oil-DHA Supplementation for Cognitive Function in Thai Children: A Randomized, Doubled-Blind, Two-Dose, Placebo-Controlled Clinical Trial. Foods.

[62] Vuholm S., Teisen M.N., Mølgaard C., Lauritzen L., Damsgaard C.T. (2021). Sleep and physical activity in healthy 8-9-year-old children are affected by oily fish consumption in the FiSK Junior randomized trial. Eur. J. Nutr..

[63] Montgomery P., Burton J.R., Sewell R.P., Spreckelsen T.F., Richardson A.J. (2014). Fatty acids and sleep in UK children: Subjective and pilot objective sleep results from the DOLAB study—A randomized controlled trial. J. Sleep Res..

[64] Richardson A.J., Burton J.R., Sewell R.P., Spreckelsen T.F., Montgomery P. (2012). Docosahexaenoic acid for reading, cognition and behavior in children aged 7–9 years: A randomized, controlled trial (the DOLAB Study). PLoS ONE.

[65] Papamichael M., Katsardis C., Tsoukalas D., Erbas B., Itsiopoulos C. (2018). A Clinical Trial of Mediterranean Diet Enriched with Fatty Fish in Pediatric Asthma: Study Protocol. J. Pharm. Pharmacol..

[66] Miles E.A., Childs C.E., Calder P.C. (2021). Long-chain polyunsaturated fatty acids (LCPUFAs) and the developing immune system: A narrative review. Nutrients.

[67] Bazinet R.P., Layé S. (2014). Polyunsaturated fatty acids and their metabolites in brain function and disease. Nat. Rev. Neurosci..

[68] Elsamman K. (2024). Polyunsaturated Fatty Acids and Traumatic Brain Injury. Nutrition and Traumatic Brain Injury (TBI) from Bench to Bedside.

[69] Joffre C., Dinel A.-L., Chataigner M., Pallet V., Layé S. (2020). N-3 polyunsaturated fatty acids and their derivates reduce neuroinflammation during aging. Nutrients.

[70] DiNicolantonio J.J., O’Keefe J.H. (2020). The Importance of Marine Omega-3s for Brain Development and the Prevention and Treatment of Behavior, Mood, and Other Brain Disorders. Nutrients.

[71] Bloch M.H., Qawasmi A. (2011). Omega-3 fatty acid supplementation for the treatment of children with attention-deficit/hyperactivity disorder symptomatology: Systematic review and meta-analysis. J. Am. Acad. Child. Adolesc. Psychiatry.

[72] Bent S., Bertoglio K., Ashwood P., Bostrom A., Hendren R.L. (2011). A pilot randomized controlled trial of omega-3 fatty acids for autism spectrum disorder. J. Autism Dev. Disord..

[73] Sonuga-Barke E.J., Brandeis D., Cortese S., Daley D., Ferrin M., Holtmann M., Stevenson J., Danckaerts M., Van der Oord S., Döpfner M. (2013). Nonpharmacological interventions for ADHD: Systematic review and meta-analyses of randomized controlled trials of dietary and psychological treatments. Am. J. Psychiatry.

[74] Chang J.P., Su K.P. (2020). Nutritional Neuroscience as Mainstream of Psychiatry: The Evidence- Based Treatment Guidelines for Using Omega-3 Fatty Acids as a New Treatment for Psychiatric Disorders in Children and Adolescents. Clin. Psychopharmacol. Neurosci..

[75] Strawn J.R., Patino R.L., Schneider M.R., DelBello M.P., McNamara R.K. (2013). Long-Chain Omega-3 Fatty Acids in Child and Adolescent Psychiatry: I. Phenomenology. Child Adolesc. Psychopharmacol. News.

[76] Richardson A.J., Montgomery P. (2005). The Oxford-Durham study: A randomized, controlled trial of dietary supplementation with fatty acids in children with developmental coordination disorder. Pediatrics.

[77] Kean J.D., Camfield D., Sarris J., Kras M., Silberstein R., Scholey A., Stough C. (2013). A randomized controlled trial investigating the effects of PCSO-524, a patented oil extract of the New Zealand green lipped mussel (*Perna canaliculus*), on the behaviour, mood, cognition and neurophysiology of children and adolescents (aged 6–14 years) experiencing clinical and sub-clinical levels of hyperactivity and inattention: Study protocol ACTRN12610000978066. Nutr. J..

[78] Giles G.E., Mahoney C.R., Kanarek R.B., Watson R.R., De Meester F. (2014). Chapter 25—Omega-3 Fatty Acids and Cognitive Behavior. Omega-3 Fatty Acids in Brain and Neurological Health.

[79] Li M., Li Z., Fan Y. (2025). Omega-3 fatty acids: Multi-target mechanisms and therapeutic applications in neurodevelopmental disorders and epilepsy. Front. Nutr..

[80] Wang Z., Feng W., Li X., Yun X., Wu S., Du L., Wang H. (2026). Targeting the Nrf2/HO-1 aixs: A therapeutic strategy against regulated cell death in Alzheimer’s disease. Ageing Res. Rev..

[81] Michael-Titus A.T., Priestley J.V. (2014). Omega-3 fatty acids and traumatic neurological injury: From neuroprotection to neuroplasticity?. Trends Νeurosci..

[82] Greenham M., Botchway E., Knight S., Bonyhady B., Tavender E., Scheinberg A., Anderson V., Muscara F. (2022). Predictors of participation and quality of life following major traumatic injuries in childhood: A systematic review. Disabil. Rehabil..

[83] de Carvalho Panzeri Carlotti A.P., do Amaral V.H., de Carvalho Canela Balzi A.P., Johnston C., Regalio F.A., Cardoso M.F., Ferranti J.F., Zamberlan P., Gilio A.E., Malbouisson L.M.S. (2025). Management of severe traumatic brain injury in pediatric patients: An evidence-based approach. Neurol. Sci..

[84] Figueroa J.D., Cordero K., Baldeosingh K., Torrado A.I., Walker R.L., Miranda J.D., De Leon M. (2012). Docosahexaenoic acid pretreatment confers protection and functional improvements after acute spinal cord injury in adult rats. J. Neurotrauma.

[85] Bailes J.E., Mills J.D. (2010). Docosahexaenoic acid reduces traumatic axonal injury in a rodent head injury model. J. Νeurotrauma.

[86] Mills J.D., Bailes J.E., Sedney C.L., Hutchins H., Sears B. (2011). Omega-3 fatty acid supplementation and reduction of traumatic axonal injury in a rodent head injury model. J. Neurosurg..

[87] Park M.-S., Oh H.-A., Ko I.-G., Kim S.-E., Kim S.-H., Kim C.-J., Kim H.-B., Kim H. (2014). Influence of mild traumatic brain injury during pediatric stage on short-term memory and hippocampal apoptosis in adult rats. J. Exerc. Rehabil..

[88] Mullen S. (2018). Major depressive disorder in children and adolescents. Ment. Health Clin..

[89] Zailani H., Wang W.L., Satyanarayanan S.K., Chiu W.C., Liu W.C., Sung Y.S., Chang J.P., Su K.P. (2024). Omega-3 polyunsaturated fatty acids and blood-brain barrier integrity in major depressive disorder: Restoring balance for neuroinflammation and neuroprotection. Yale J. Biol. Med..

[90] Borsini A., Nicolaou A., Camacho-Muñoz D., Kendall A.C., Di Benedetto M.G., Giacobbe J., Su K.-P., Pariante C.M. (2021). Omega-3 polyunsaturated fatty acids protect against inflammation through production of LOX and CYP450 lipid mediators: Relevance for major depression and for human hippocampal neurogenesis. Mol. Psychiatry.

[91] Echeverría F., Valenzuela R., Hernandez-Rodas M.C., Valenzuela A. (2017). Docosahexaenoic acid (DHA), a fundamental fatty acid for the brain: New dietary sources. Prostaglandins Leukot. Essent. Fat. Acids.

[92] Bazan N.G. (2005). Neuroprotectin D1 (NPD1): A DHA-derived mediator that protects brain and retina against cell injury-induced oxidative stress. Brain Pathol..

[93] Juárez-López C., Klünder-Klünder M., Madrigal-Azcárate A., Flores-Huerta S. (2013). Omega-3 polyunsaturated fatty acids reduce insulin resistance and triglycerides in obese children and adolescents. Pediatr. Diabetes.

[94] Kerling E.H., Hilton J.M., Thodosoff J.M., Wick J., Colombo J., Carlson S.E. (2019). Effect of Prenatal Docosahexaenoic Acid Supplementation on Blood Pressure in Children With Overweight Condition or Obesity: A Secondary Analysis of a Randomized Clinical Trial. JAMA Netw. Open.

[95] López-Alarcón M., Martínez-Coronado A., Velarde-Castro O., Rendón-Macías E., Fernández J. (2011). Supplementation of n3 long-chain polyunsaturated fatty acid synergistically decreases insulin resistance with weight loss of obese prepubertal and pubertal children. Arch. Med. Res..

[96] Burrows T., Collins C., Garg M. (2011). Omega-3 index, obesity and insulin resistance in children. Int. J. Pediatr. Obes..

[97] Musazadeh V., Mahmoudinezhad M., Pam P., Brazandeh S., Faramarzi F., Mohammadpour Y., Faghfouri A.H., Gheibi S. (2025). Omega-3 supplementation and cardiometabolic risk factors in obese/overweight children and adolescents: A GRADE assessed systematic review and meta-analysis. Nutr. Metab..

[98] Curioni C.C., Alves N.N.R., Zago L. (2019). Omega-3 supplementation in the treatment of overweight and obese children and adolescents: A systematic review. J. Funct. Foods.

[99] Bonafini S., Antoniazzi F., Maffeis C., Minuz P., Fava C. (2015). Beneficial effects of ω-3 PUFA in children on cardiovascular risk factors during childhood and adolescence. Prostagl Other Lipid Mediat..

[100] Ferreira C.R., van Karnebeek C.D.M. (2019). Inborn errors of metabolism. Handb. Clin. Neurol..

[101] Delpino F.M., Figueiredo L.M., da Silva B.G.C. (2021). Effects of omega-3 supplementation on body weight and body fat mass: A systematic review. Clin. Nutr. ESPEN.

[102] (2015). The 12th World Congress on Inflammation. Inflamm. Res..

[103] Poggioli R., Hirani K., Jogani V.G., Ricordi C. (2023). Modulation of inflammation and immunity by omega-3 fatty acids: A possible role for prevention and to halt disease progression in autoimmune, viral, and age-related disorders. Eur. Rev. Med. Pharmacol. Sci..

[104] Sekikawa A., Cui C., Sugiyama D., Fabio A., Harris W.S., Zhang X. (2019). Effect of High-Dose Marine Omega-3 Fatty Acids on Atherosclerosis: A Systematic Review and Meta-Analysis of Randomized Clinical Trials. Nutrients.

[105] Capra M.E., Stanyevic B., Giudice A., Monopoli D., Decarolis N.M., Esposito S., Biasucci G. (2023). Long-chain polyunsaturated fatty acids effects on cardiovascular risk in childhood: A narrative review. Nutrients.

[106] Boukid F., Castellari M. (2021). Food and Beverages Containing Algae and Derived Ingredients Launched in the Market from 2015 to 2019: A Front-of-Pack Labeling Perspective with a Special Focus on Spain. Foods.

[107] Ryan A.S., Astwood J.D., Gautier S., Kuratko C.N., Nelson E.B., Salem N. (2010). Effects of long-chain polyunsaturated fatty acid supplementation on neurodevelopment in childhood: A review of human studies. Prostaglandins Leukot. Essent. Fat. Acids.

[108] (2012). EFSA Panel on Dietetic Products, N. and Allergies, Scientific Opinion on the Tolerable Upper Intake Level of eicosapentaenoic acid (EPA), docosahexaenoic acid (DHA) and docosapentaenoic acid (DPA). EFSA J..

[109] Organisms E.P.o.G.M., Naegeli H., Bresson J.L., Dalmay T., Dewhurst I.C., Epstein M.M., Firbank L.G., Guerche P., Hejatko J., Moreno F.J. (2021). Statement complementing the EFSA Scientific Opinion on application (EFSA-GMO-NL-2010-85) for authorisation of food and feed containing, consisting of and produced from genetically modified soybean MON 87769× MON 89788. EFSA J.

[110] EFSA Panel on Dietetic Products, Nutrition, and Allergies (NDA) (2010). Scientific opinion on dietary reference values for fats, including saturated fatty acids, polyunsaturated fatty acids, monounsaturated fatty acids, trans fatty acids, and cholesterol. EFSA J..

[111] Agostoni C., Buonocore G., Carnielli V.P., De Curtis M., Darmaun D., Decsi T., Domellöf M., Embleton N.D., Fusch C., Genzel-Boroviczeny O. (2010). EESPGHAN Committee on Nutrition. Enteral nutrient supply for preterm infants: Commentary from the European Society of Paediatric Gastroenterology, Hepatology and Nutrition Committee on Nutrition. J. Pediatr. Gastroenterol. Nutr..

[112] Badoni P., Nazir I., Aier M., Maity P.B., Samanta S., Das A. (2021). Significant role of fish nutrients with special emphasis to essential fatty acid in human nutrition. Int. J. Curr. Microbiol. Appl. Sci..

[113] Vianna G.M., Zeller D., Pauly D. (2020). Fisheries and policy implications for human nutrition. Curr. Environ. Health Rep..

[114] Lenihan-Geels G., Bishop K.S., Hegde M.V. (2025). Alternative Origins for Omega-3 Fatty Acids in the Diet. Omega-3 Fatty Acids: Keys to Nutritional Health and Disease.

[115] Calder P.C. (2010). Omega-3 fatty acids and inflammatory processes. Nutrients.

[116] Schuchardt J.P., Hahn A. (2013). Bioavailability of long-chain omega-3 fatty acids. Prostaglandins Leukot. Essent. Fat. Acids.

[117] Gil A., Gil F. (2015). Fish, a Mediterranean source of n-3 PUFA: Benefits do not justify limiting consumption. Br. J. Nutr..

[118] Koletzko B., Uauy R., Palou A., Kok F.J., Hornstra G., Eilander A., Moretti D., Osendarp S., Zock P., Innis S. (2010). Dietary intake of eicosapentaenoic acid (EPA) and docosahexaenoic acid (DHA) in children—A workshop report. Br. J. Nutr..

[119] Vergara Nieto Á.A., Díaz A.H., Hernández Millán M., Sagredo D. (2025). Molecular Features, Effective Sources, and Physiological Effects of Omega-3 Unsaturated Fatty Acids on Cardiovascular, Neurological, and Muscular Health, and Clinical Relevance for Several Conditions: A Narrative Review. Nutr. Rev..

[120] Mohan D., Mente A., Dehghan M., Rangarajan S., O’Donnell M., Hu W., Dagenais G., Wielgosz A., Lear S., Wei L. (2021). Associations of fish consumption with risk of cardiovascular disease and mortality among individuals with or without vascular disease from 58 countries. JAMA Intern. Med..

[121] Smolińska K., Szopa A., Sobczyński J., Serefko A., Dobrowolski P. (2024). Nutritional Quality Implications: Exploring the Impact of a Fatty Acid-Rich Diet on Central Nervous System Development. Nutrients.

[122] Joint F., Consultation W.E. (2008). Fats and fatty acids in human nutrition. Rep. Expert. Consult..

[123] Lewin G.A., Schachter H.M., Yuen D., Merchant P., Mamaladze V., Tsertsvadze A. (2005). Effects of omega-3 fatty acids on child and maternal health. Evid. Rep. Technol. Assess. (Summ.).

[124] Page M.J., McKenzie J.E., Bossuyt P.M., Boutron I., Hoffmann T.C., Mulrow C.D., Shamseer L., Tetzlaff J.M., Akl E.A., Brennan S.E. (2021). The PRISMA 2020 statement: An updated guideline for reporting systematic reviews. BMJ.

[125] Grossman J., Mackenzie F.J. (2005). The randomized controlled trial: Gold standard, or merely standard?. Perspect. Biol. Med..

